# Adrenal Function in Females with Low Plasma HDL-C Due to Mutations in *ABCA1* and *LCAT*

**DOI:** 10.1371/journal.pone.0090967

**Published:** 2014-05-19

**Authors:** Andrea E. Bochem, Adriaan G. Holleboom, Johannes A. Romijn, Menno Hoekstra, Geesje M. Dallinga, Mahdi M. Motazacker, G. Kees Hovingh, Jan A. Kuivenhoven, Erik S. G. Stroes

**Affiliations:** 1 Department of Vascular Medicine, Academic Medical Center, Amsterdam, The Netherlands; 2 Department of Medicine, Academic Medical Center, Amsterdam, The Netherlands; 3 Leiden/Amsterdam Center for Drug Research, Leiden, The Netherlands; 4 Department of Experimental Vascular Medicine, Academic Medical Center, Amsterdam, The Netherlands; 5 Department of Pathology & Medical Biology, Medical Biology Section, Molecular Genetics, University Medical Center Groningen, University of Groningen, The Netherlands; Harvard Medical School, United States of America

## Abstract

**Introduction:**

Adrenal steroidogenesis is essential for human survival and depends on the availability of the precursor cholesterol. Male subjects with low plasma levels of high density lipoprotein (HDL) cholesterol are characterized by decreased adrenal function. Whether this is also the case in female subjects with low plasma HDL-C levels is unresolved to date.

**Findings:**

15 female ATP binding cassette transporter AI (*ABCAI*) and 14 female lecithin-cholesterol acyltransferase (*LCAT*) were included in the study. HDL-C levels were 38% and 41% lower in *ABCA1* and *LCAT* mutation carriers compared to controls, respectively. Urinary steroid excretion of 17-ketogenic steroids or 17-hydroxy corticosteroids did not differ between 15 female *ABCA1* mutation carriers (p = 0.27 vs 0.30 respectively) and 30 matched normolipidemic controls or between 14 female *LCAT* mutation carriers and 28 matched normolipidemic controls (p = 0.10 and 0.14, respectively). Cosyntropin testing in an unselected subgroup of 8 *ABCA1* mutation carriers and 3 *LCAT* mutation carriers did not reveal differences between carriers and controls.

**Conclusion:**

Adrenal function in females with molecularly defined low HDL-C levels is not different from controls. The discrepancy with the finding of impaired steroidogenesis in males with molecularly defined low HDL-C levels underscores the importance of gender specific analyses in cholesterol-related research.

## Introduction

The adrenal gland plays a pivotal role in essential physiological processes such as the regulation of stress response, blood pressure and electrolyte homeostasis. Cholesterol is the substrate for adrenal steroidogenesis and is for 75% derived from plasma lipoproteins [1], [2]. However, whether the availability of lipoprotein derived cholesterol is a rate-limiting factor for adrenal steroidogenesis in humans is sparsely investigated to date.

The importance of low density lipoprotein (LDL) derived cholesterol for adrenal steroidogenesis has been studied in patients with familial hypercholesterolemia (FH), carrying mutations in the LDL receptor (*LDLR)*. The adrenal gland takes up plasma cholesterol via endocytosis of the LDL receptor [3]. In line with this notion, adrenal steroidogenesis is impaired in homozygous *LDLR* mutation carriers [4] and homozygous apolipoprotein B (*APOB*) mutation carriers, suffering from genetically impaired binding of LDL to the LDL receptor [5], [6], [7]. Heterozygous *LDLR* and *APOB* carriers, however, did not show any signs of impaired adrenal steroidogenesis [5], [8]. Together, these studies indicate that plasma lipoprotein derived cholesterol plays a role in adrenal steroidogenesis, but that LDL derived cholesterol does not constitute the major source of substrate for the adrenal glands.

High density lipoprotein (HDL) has been suggested to be the preferred lipoprotein for cholesterol delivery to the adrenal gland in adrenal cell lines [9] and in murine models [10], [11], [12], [13], [14], [15]. In critically ill patients, low HDL-C levels are associated with impaired adrenal responses to synthetic ACTH [16]. Furthermore, others reported a high incidence of adrenal failure in critically ill individuals, with HDL-C being the only variable predictive of adrenal insufficiency [17].

Importantly, adrenal function was assessed in individuals with functional mutations in *SCARB1*, the gene encoding the HDL receptor Scavenger Receptor B1, which is highly expressed on the adrenal gland. Heterozygous *SRB1* mutation carriers display markedly decreased adrenal steroidogenesis [18]. Furthermore, we recently demonstrated that male individuals with low plasma HDL-C levels are characterized by decreased adrenal function [19], further underlining the importance of HDL derived cholesterol for adrenal steroidogenesis. However, whether adrenal steroidogenesis is also impaired in females with low plasma HDL-C remains to be investigated.

We set out to assess adrenal function in female carriers of functional mutations in ATP-binding cassette transporter A1 (*ABCA1*) and lecithin-cholesterol acyltransferase (*LCAT*), typically displaying half-normal plasma levels of HDL-C. We hypothesized that in female subjects with low HDL-C levels, adrenal function is compromised irrespective of the molecular origin of the low HDL-C levels.

## Methods

### Recruitment of Study Participants

Subjects with HDL-C levels <5^th^ percentile were screened for mutations in *ABCA1* and *LCAT* [20], [21]. Data were obtained in parallel with a study on adrenal function in male *ABCA1* and *LCAT* mutation carriers [19]. For the current study, we enrolled 15 female carriers of mutations in *ABCA1* and 14 female carriers of mutations in the *LCAT* gene. Functionality of all mutations was established in previously published studies [21], [22], [23]. As a control group, normolipidemic age matched female individuals were recruited by advertisement. In order to increase power, carriers were matched to controls in a 1:2 fashion. None of the included individuals used oral contraceptives or medication interfering with steroid metabolism. The study was approved by the institutional review board of the Academic Medical Center, Amsterdam, The Netherlands. All participants provided written informed consent.

### Questionnaire and Biochemical Measurements

Medical history, cardiovascular risk factors and use of medication were assessed using a questionnaire. Brachial artery blood pressures was measured using an oscillometric blood pressure device (Omron 705IT, Hoofddorp, the Netherlands). Hypertension was defined as 1) use of antihypertensive medication and/or 2) a systolic blood pressure at visit above 140 mmHg and/or diastolic blood pressure above 90 mmHg.

Plasma was obtained after an overnight fast and stored at −80°C. Total cholesterol, LDL-C, HDL-C and triglyceride levels were analyzed using commercially available enzymatic methods (Randox, Antrim, United Kingdom and Wako, Neuss, Germany) on a Cobas Mira autoanalyzer (Roche, Basel, Switzerland). Aldosterone was measured using a radioimmunoassay (Siemens, Los Angeles, USA).

### Baseline Adrenal Steroidogenesis

All study participants followed the same protocol to collect 24-hour urine: on day 1, the first morning urine is not collected. After the morning urine, all urine is collected in the container. Participants collected urine for 24 hours, and ended with adding the morning urine of day 2 to the container. Urinary excretion of steroid metabolites was analyzed by gas chromatography in 24-hour urine samples as previously described [24], [25]. Androsteron (A), etiocholanolon (E), dehydroepiandrosteron (D), 11-keto-androsteron (KA), 11-keto-etiocholanolon (KE), 11-hydroxy-androsteron (HA), 11-hydroxy-etiocholanolon (HE), pregnaandiol (P2), Pregnaantriol (P3), 11-deoxytetrahydrocortisol (THS), tetrahydrocortison (THE), tetrahydrocortisol (THF) and allo-tetrahydrocortisol (ALLO) were measured as readout of adrenal steroidogenesis. A, E, D, KA, KE, HA and HE make up total 17-ketogenic steroids (17-KS), whereas THS, THE, THF, ALLO and P3 are the constituents of total 17-hydroxycorticoids (17-OHCS). In addition, urinary free cortisol was determined using solid-phase extraction-liquid chromatography- tandem mass spectrometry on a Symbiosis Pharma (Spark Holland, Emmen, The Netherlands) Quattro premier Tandem Mass spectrometer (Waters, Millford MA) system. Solid Phase extraction was performed on Oasis HLB cartridges (Waters, Millford, MA), chromatographic separation was achieved on a Waters Sunfire C18 column 3.5 µm 2.1×50 mm using ammonium acetate mM with 0.1% formic acid as mobile phase and acetonitrile as mobile phase B. Limit of detection 5 nmol/L, intra-assay variation <4%, total assay variation <7%.

### Stimulated Adrenal Steroidogenesis

Unselected subgroups of 8 *ABCA1* and 3 *LCAT* mutation carriers consented to an ACTH stimulation study (co-syntropin or tetracosactin, 0.25 mg/ml, Novartis Pharma b.v., Arnhem, The Netherlands). All the cosyntropin tests started strictly at 9.00 am. All patients underwent an overnight fast prior to cosyntropin testing. Two baseline blood samples were obtained, 15 minutes and 1 minute before administration of the 1 µg cosyntropin bolus. Subsequent blood samples were drawn 30 minutes and 60 minutes after cosyntropin administration. Plasma cortisol levels were measured by enzyme immunoassay (Siemens Medical Solutions, Los Angeles, CA), and cortisol-binding globulin (CBG) levels were measured with a commercial radioimmunoassay (Siemens Medical Solutions, Los Angeles, CA). Free cortisol levels were calculated using the method described by Coolens et al [26].

### Statistical Analysis

Unpaired student’s T-test was performed for analysis of continuous data with a normal distribution. In case of a skewed distribution, data were log-transformed prior to T-testing. Categorical data were assessed by χ^2^-testing. A p-value of <0.05 was considered statistically significant. Interaction analyses were performed to establish the gender specific effect of carriership on adrenal steroidogenesis.

## Results

### Population Characteristics

We enrolled 15 and 14 female carriers of loss of function mutations in *ABCA1* and *LCAT,* respectively. Three of the *ABCA1* mutation carriers were either compound heterozygous or homozygous, while two of the *LCAT* mutation carriers were homozygous. None of the participants was referred to our clinic for symptoms of adrenal dysfunction. Age-matched female family members were asked to participate as controls. As an insufficient number of family members volunteered, unrelated age-matched female controls were recruited by advertisement. Demographic, clinical and biochemical characteristics of all study participants are listed in table 1. As expected, HDL-C levels were 38% lower in carriers of *ABCA1* mutations and 41% lower in *LCAT* mutation carriers, compared to normolipidemic controls (p<0.001). Hypertension was more prevalent in *ABCA1* mutation carriers (p = 0.002) and *LCAT* mutation carriers (p = 0.02). Systolic and diastolic blood pressure was significantly higher in *LCAT* mutation carriers compared to controls (p = 0.03 and p = 0.03, respectively). Other parameters did not differ significantly.

**Table 1:** Characteristics of female ATP-binding cassette transporter 1 *(ABCA1*) and Lecithin-cholesterol acyltransferase (*LCAT*) mutation carriers and matched female controls.

Characteristics	*ABCA1*			*LCAT*		
	*Controls (n = 30)*	*Carriers (n = 15)*	*p value*	*Controls (n = 28)*	*Carriers (n = 14)*	*P value*
**Demographic**						
Age - yrs	48.1±14.8	47.4±14.5	0.88	41.4±17.4	41.7±15.2	0.99
BMI (kg/m^2^)	24.8±4.0	25.7±7.4	0.59	24.7±4.3	25.1±5.9	0.79
Current smokers - no (%)	4 (13)	2 (13)	1.00#	3 (10)	1 (25)	0.74#
Statin users - no (%)	3 (33)	6 (67)	0.10#	3 (38)	5 (63)	0.25#
Alcohol users - no (%)	19 (63)	8 (53)	0.52#	18 (62)	7 (50)	0.45#
**Clinical**						
Coronary artery disease - no (%)	1 (3)	4 (27)	∼	1 (3)	0	∼
Diabetes mellitus - no (%)	0	1 (7)	∼	0	0	∼
Hypertension - no (%)	2 (7)	7 (47)	0.002#	1 (3)	4 (29)	0.02#
Systolic blood pressure (mmHg)	121.1±16.9	136.1±24.5	0.07	117.0±10.1	130.2±16.6	0.03
Diastolic blood pressur (mmHg)	73.0±11.7	80.0±10.2	0.10	71.9±10.2	81.1±10.2	0.03
**Biochemical**						
Aldosterone (nmol/L)	0.17±0.15	0.26±0.22	0.23	0.18±0.15	0.17±0.14	0.88
Cortisol Binding Globulin (mg/L)	68.0±40.8	65.3±2.5	0.86	68±40.8	73.3±41.3	0.85
Total cholesterol (mmol/L)	4.50±1.10	4.41±1.47	0.14	4.82±1.12	4.35±1.10	0.21
LDL cholesterol (mmol/L)	3.23±1.03	3.20±1.08	0.95	3.07±1.07	3.04±0.81	0.94
HDL cholesterol (mmol/L)	1.43±0.34	0.88±0.51	<0.001	1.48±0.36	0.87±0.40	<0.001
Triglycerides mmol/L	0.89 (0.65–1.28)	1.09 (0.71–1.63)	0.25	0.80 (0.60–1.21)	1.12 (0.68–1.64)	0.10

Values are means ± SD unless otherwise indicated. Triglycerides are median with interquartile range. P for Student’s T-test. Triglycerides were logtransformed prior to T-test.

# P for χ^2^ test. No t-test was performed for history of coronary artery disease since referral bias was present. Partial overlap exists between the two control cohorts.

The significance of the interaction terms allows us to separately assess adrenal steroidogenesis in male and female carriers.

### Basal Adrenal Steroidogenesis

**Figure 1:**
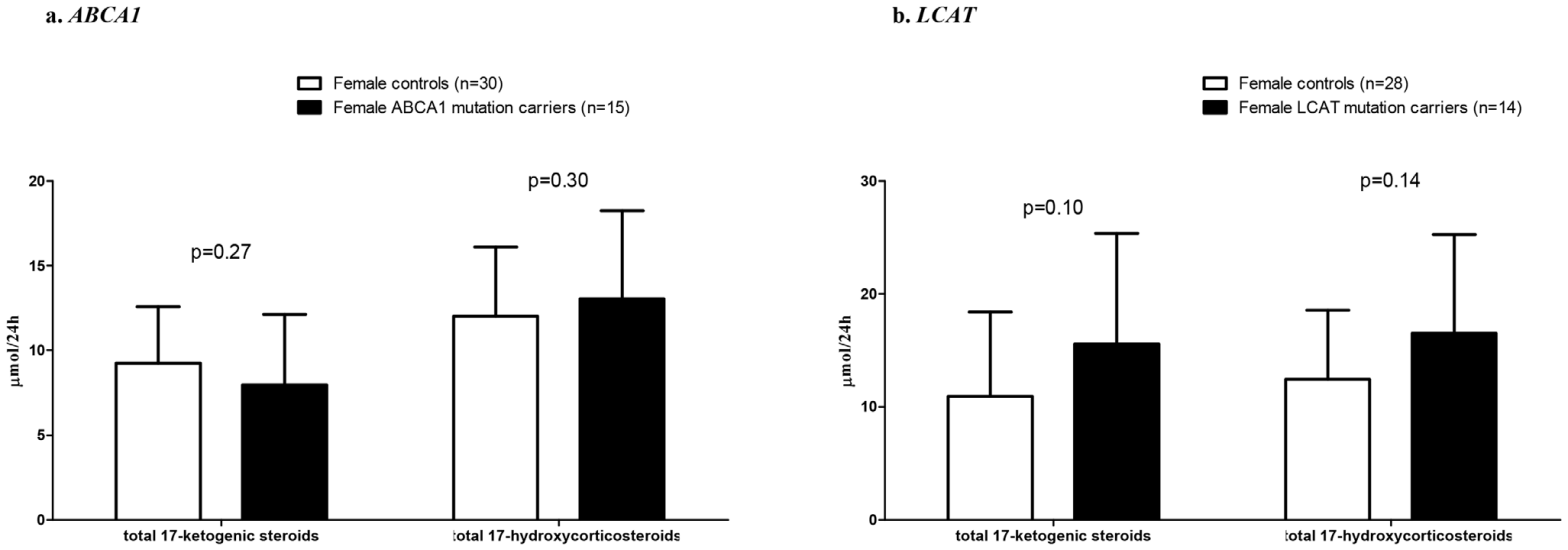
24-hour urinary steroid excretion in male *ABCA1* and *LCAT* mutation carriers compared to age-matched female controls. Data are presented as mean ± SD. P values for student’s t-test.

**Figure 2:**
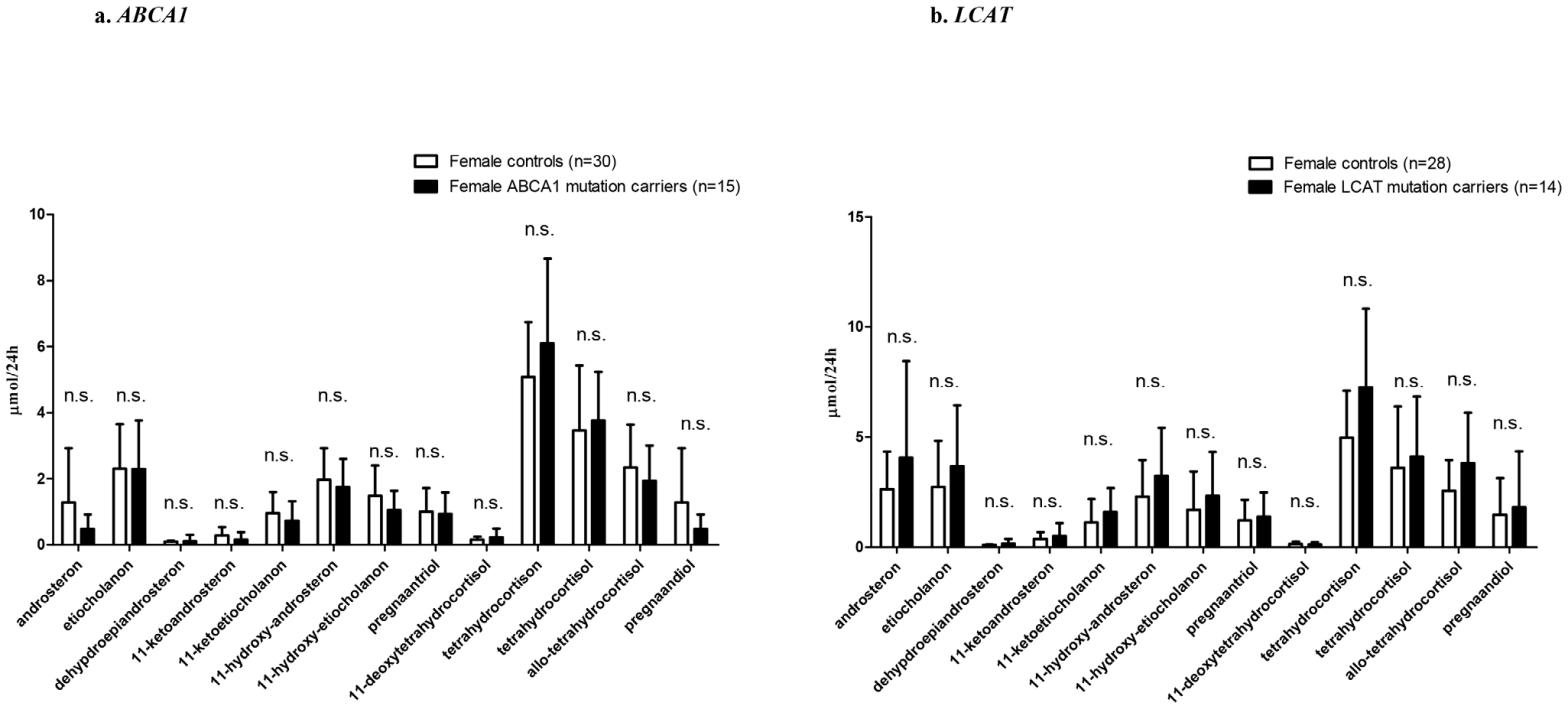
Urinary steroid metabolites in female *ABCA1* and *LCAT* mutation carriers compared to age-matched female controls. Data are presented as mean ± SD. P values for student’s t-test.

Twenty four hour urinary excretion of 17-ketogenic steroids (17-KS) or 17-hydroxycorticosteroids (17-OHCS) did not differ between carriers of mutations in *ABCA1* and controls (p = 0.27 and 0.30, Figure 1a), or between *LCAT* mutation carriers and controls (p = 0.10 and 0.14, Figure 1b). Urinary steroid excretions were within the normal range for both for 17-KS and 17-OHCS [25]. The full panel of urinary steroid metabolites is presented in figure 2a–b.

No gene-dose effect was observed when comparing the three compound heterozygous/homozygous *ABCA1* mutation carriers or the two homozygous *LCAT* mutation carriers to heterozygous carriers and controls.

### Adrenal Response to Cosyntropin

**Figure 3:**
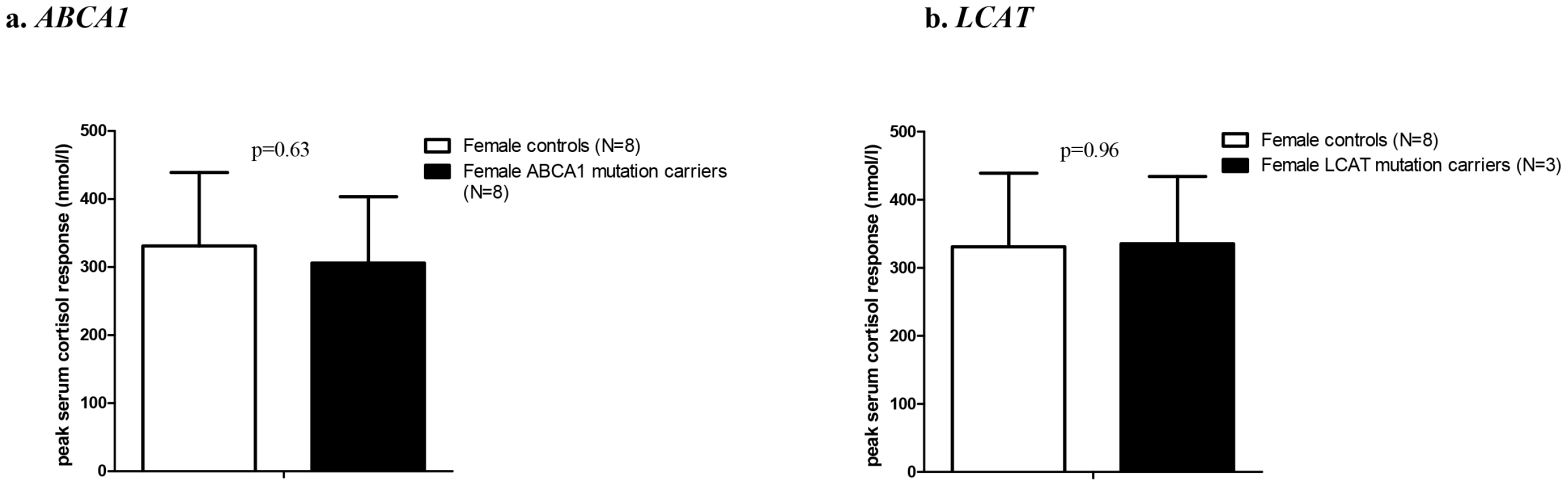
Peak serum cortisol increase after cosyntropin administration in female *ABCA1* and *LCAT* mutation carriers versus female controls. Data are presented as mean ± SD. P values for Student’s T-test.

In a 1 µg cosyntropin stimulation test, the cortisol response to physiological levels of ACTH is measured as a proxy of adrenal cortical function [27], [28]. The peak serum cortisol response to ACTH was not different between *ABCA1* and *LCAT* mutation carriers and did not differ from controls (p = 0.63 and 0.96, respectively, figure 3a–b). In addition, peak plasma levels of free cortisol, taking into account possible differences in cortisol binding globulin (CBG) levels [18], were not different. Plasma lipid levels did not differ significantly before and after cosyntropin testing (data not shown).

## Discussion

This study shows that adrenal steroidogenesis is not impaired in female individuals with low plasma HDL-C levels. This is in contrast with our earlier finding of lower basal urinary steroid excretion in males with low plasma HDL-C levels. These findings underline the differences between men and women in terms of cholesterol metabolism and adrenal steroidogenesis and emphasize the importance of gender-specific analyses in cholesterol related research.

Studies showing decreased adrenal steroidogenesis in murine models of compromised availability of HDL derived cholesterol were carried out in both males [13], [14] and females [12]. These studies did not report gender specific effects [12], [13], [14]. It should be taken into account, however, that cholesterol metabolism differs greatly between mice and men and, as a consequence, results derived from murine models are not necessarily reflecting human (patho)physiology. Human studies addressing this question are sparse and data about the distribution of males and females are lacking [18]. Interaction analyses, however, established a gender specific effect of carriership on adrenal steroidogenesis. This is in line with the fact that the reference values of our parameters are gender specific [24], [25]. Furthermore, no articles on the role of cholesterol in adrenal steroidogenesis pool data of males and females [4], [5], [6], [7], [8].

Our findings constitute the first evidence of differential adrenal cholesterol handling in males and females. Based on the current data, we can only speculate about the mechanism underlying the differential effect of low plasma HDL-C levels in men and women. In murine models, there was no differential effect between male and female animals, and, therefore, experiments in these experimental models can not be expected to elucidate the gender-dependent findings in humans.

Plasma HDL cholesterol levels are gender specific, as reflected by different reference values for men and women [29]. Furthermore, the correlation between HDL-C levels with age is negative in women, but positive in men [30]. The correlation between HDL-C levels and cardiovascular disease has been described to be gender specific. For example, plasma levels of LCAT were associated with low HDL-C levels in men, but not in women. Moreover, plasma LCAT levels were associated with a surprisingly increased CHD risk in women, but not in men [31].

Gender-specific effects in adrenal function have also been described. Different reference values are used for urinary steroidogenesis in males and females [25]. Furthermore, females have been shown to exhibit a stronger response to synthetic ACTH than males [32]. However, neither explains the gender-specific findings in our cohort.

Several mechanisms may explain the different effects of low HDL cholesterol levels on adrenal steroidogenesis between men and women. First, women are characterized by higher levels of estradiol compared to men, which has shown to be essential in maintaining an adequate adrenal output [33]. This may constitute an additional stimulatory pathway, which men lack, equipping women with sufficient adrenal stimuli to overcome the effects of low availability of substrate. Conversely, men display higher levels of androgens, which have shown to be associated with decreased adrenal function [34], indicating that men not only lack the stimulatory effects of estrogen on adrenal steroidogenesis, but have an inhibitory pathway instead. On top of low levels of HDL-derived cholesterol, this may compromise adrenal steroidogenesis in males, whereas females are relatively protected.

Furthermore, the gender-specific differential effects may pertain to differences in the immune system. Interleukin (IL) 6 is essential for the activation of the hypothalamic-pituitary-adrenal axis [35], [36]. IL6 expression is lower in males compared to females [37]. Given the fact that male adrenal steroidogenesis is more strongly affected by plasma interleukin 6 (IL6) than female adrenal steroidogenesis [38], this additional lack of adrenal stimulation in low HDL males, may tip the balance to lower adrenal steroidogenesis in males, whereas females both have higher IL6 expression and less dependence of adrenal steroidogenesis on IL6 levels.

Interestingly, hypertension was more prevalent in both *ABCA1* and *LCAT* mutation carriers, but seemed better regulated in *ABCA1* mutation carriers. Increased prevalence of hypertension has been previously reported in *LCAT* mutation carriers [39]. Given the absence of a difference in both plasma aldosterone levels and adrenal function, an adrenal component is unlikely to contribute to the increased hypertension in carriers.

A limitation of this study is the fact that moment in menstrual cycle was not recorded, nor was pre- or postmenopausal state. However, by closely matching study participants for age, the chance was minimized that these age-related circumstances influenced results. Furthermore, the study cohort was relatively small, inherent to studies in subjects with rare mutations. However, false-negative findings are unlikely given the small differences between carriers and non-carriers: −1.29 ηmol/24 h for total 17-ketogenic steroids and 1.02 ηmol/24 h for total 17-hydroxycorticoids. For comparison, the difference in male carriers compared to controls was 6.79 ηmol/24 h for total 17-ketogenic steroids and 4.85 ηmol/24 h for total 17-hydroxycorticoids.

Alltogether, our data show that adrenal function in females with molecular defined low HDL-C levels is not different from controls. The discrepancy with the finding of impaired steroidogenesis in males with molecularly defined low HDL-C levels, underscores the importance of gender specific analyses in cholesterol related research.
